# Nanoparticles Targeting the Molecular Pathways of Heart Remodeling and Regeneration

**DOI:** 10.3390/pharmaceutics14040711

**Published:** 2022-03-26

**Authors:** Diana Gonciar, Teodora Mocan, Lucia Agoston-Coldea

**Affiliations:** 1 2nd Department of Internal Medicine, Faculty of Medicine, “Iuliu Hațieganu” University of Medicine and Pharmacy, Cluj-Napoca 400000, Romania; dianagonciar@gmail.com (D.G.); luciacoldea@yahoo.com (L.A.-C.); 2 Physiology Department, Faculty of Medicine, “Iuliu Hațieganu” University of Medicine and Pharmacy, Cluj-Napoca 400000, Romania; 3 Department of Nanomedicine, Regional Institute of Gastroenterology and Hepatology, Cluj-Napoca 400162, Romania

**Keywords:** nanoparticles, remodeling, regeneration, cardiomyocytes

## Abstract

Cardiovascular diseases are the main cause of death worldwide, a trend that will continue to grow over the next decade. The heart consists of a complex cellular network based mainly on cardiomyocytes, but also on endothelial cells, smooth muscle cells, fibroblasts, and pericytes, which closely communicate through paracrine factors and direct contact. These interactions serve as valuable targets in understanding the phenomenon of heart remodeling and regeneration. The advances in nanomedicine in the controlled delivery of active pharmacological agents are remarkable and may provide substantial contribution to the treatment of heart diseases. This review aims to summarize the main mechanisms involved in cardiac remodeling and regeneration and how they have been applied in nanomedicine.

## 1. Introduction

Ischemic heart disease is the main cause of death worldwide, with more than 9 million deaths annually, as reported by the World Health Organization [1]. The applications of nanomedicine in cardiovascular pathology are still in their infancy, with 1702 published articles on this topic in the last 10 years according to Scopus.

The most currently available drugs used in the management of cardiovascular diseases lack the ability to target the damaged tissue, thereby reaching low concentration and significant damage to other tissues [2]. An important component of cardiovascular diseases is atherosclerosis, which may result in localized reduced blood flow and impaired drug or contrast agent delivery at the area of interest [3]. In end-stage disease, the only therapeutic solution is represented by heart transplantation, which is limited due to the reduced number of donors, making the need to develop novel regenerative strategies more conspicuous. The main advantages of nanoformulations are the protection of the drugs from degradation and targeted delivery with reduced side effects [4]. The cargo may be loaded by encapsulation or by surface attachment and it is even possible to deliver hydrophilic and hydrophobic agents simultaneously [5]. 

The factors that control the distribution of the nanostructures are the size, shape, surface changes, to cite only a few. In general, nanoparticles greater than 1000 nm tend to accumulate into the liver and lung, the ones smaller than 8 nm are eliminated by ultrafiltration, while nanomaterials between 10 and 300 nm accumulate in organs with high-density of macrophages (spleen, lymph nodes, bone marrow, and liver) [6]. When introduced to the body, the immune system interacts with the nanoparticles in the attempt to isolate and remove them. In this direction, the use of synthetic coating emerged, such as polyethylene glycol (PEG), prolonging the half-life in blood [5]. There is increasing evidence for the administration of nanoparticles for the diagnosis and treatment of ischemic heart disease, especially because they are more likely to be taken up by cells, thereby having a low risk of embolization [7]. Intravenous injection is the preferable path of administration because it is less invasive than intramyocardial delivery. In myocardial infarction, due to the formation of granulation tissue, the passive accumulation of the nanoparticles is facilitated by enhanced permeability and retention effect [7]. However, this effect was reported to decline after 24–48 h after myocardial infarction, implying a narrow window to administrate the pristine nanoformulations [8]. Intramyocardial administration, although appropriate in an acute context, is not indicated in myocardial infarction, given the friability of the granulation tissue, which brings an increased risk of rupture of the heart wall [7]. While the oral route is the preferred way in terms of compliance, the absorption is sometimes unsatisfactory in the case of cardiovascular diseases [9]. The inhalation route is suitable in chronic conditions and Miragoli et al. [10] reported the usage of calcium phosphate nanoparticles in this regard, offering the benefit of non-invasiveness and effective delivery by reduced first-pass metabolism. However, this route is limited in the case of lung diseases, which are important causes of right-sided heart remodeling. 

The main classes of nanoparticles are: polymeric, lipid-based, and inorganic [11], each with potential application in cardiovascular diseases. Polymeric nanoparticles are good drug-delivery platforms via oral or intravenous administration routes [12] due to their biocompatibility, versatility, and stability offering multiple modalities to load the desired drugs: by encapsulating, conjugating to the polymer, embedding in the matrix, or attaching them to the surface [11]. Polymeric nanoparticles are designed as nanocapsules, with a central core and a polymeric shell or as nanospheres, having a solid polymeric matrix. They are further divided according to their shape in polymersomes, micelles, and dendrimers [11]. Their main limitation is high particle aggregation. Polymeric nanoparticles are made up by natural polysaccharides (hyaluronic acid, cellulose, and chitosan), proteins (albumin, elastin, and collagen) or synthetic polymers (polyesters, polyanhydrides, and polyamines) [13]. Due to their potential to protect the payload, increase the half-time in the plasma and reduce the side effects, there are multiple applications in the field of cardiology that will be later detailed.

Liposomes are spherical structures composed by an aqueous central core, surrounded by one or multiple lipid bilayers, able to be loaded with hydrophilic drugs in the inner compartment and with lipophilic agents in the lipid bilayer [14]. They are useful for both drug delivery and imaging applications and may also be used as direct treatments, being able to seal the injured endothelium [3]. The uptake of liposomes in the cardiac muscle cells remains low, compared to the monocyte–macrophage system, being negatively influenced by opsonization. Through electrostatic interaction between the positively charged liposomes and negatively charged cellular membranes, the nanoparticles are internalized into the cell and release the payload [15]. The state of the tissue is another important determinant of their distribution: cationic liposomes accumulate preferentially in the diseased myocardium, in areas with granulation tissue and chronic inflammation [16]. The downsides of liposomes are the rapid clearance (ameliorated by PEG coating) and possible immunological reactions [3]. 

Solid lipid nanoparticles offer a solid matrix at room and body temperature that provides a stable environment for the controlled release of both hydrophilic and lipophilic drugs by protection against degradation from storage time, metabolism, or elimination [17]. The lipid matrix is made up by biocompatible, nontoxic lipids (highly purified glycerides, glyceride mixtures, and waxes). They are promising carriers for oral administration and have multiple pharmaceutical forms: capsules, tablets, and pellets. Solid lipid nanoparticles manufactured by palmitic acid, poloxamer 188, and soy lecithin were reported to improve the oral bioavailability of nimodipine (calcium channel blocker) by increased adherence to the mucosal lining of the gastrointestinal tract and high lymphatic uptake [18]. Lipid-based nanoparticles undergo lipolysis at sites with high lipase amount, especially in the small bowel, being transformed in micelles or can be translocated uncleaved [19]. The lipolysis may also take place intracellularly by lysosomal acid lipase [19]. 

In addition to therapeutic solutions, nanomedicine also finds its role in cardiovascular imaging, with applications in the early detection of lesions, from a preclinical stage. Adverse cardiac remodeling is associated with poor clinical outcomes; thus, it is an important parameter to be monitored [20]. The imaging methods commonly used in cardiovascular disease include magnetic resonance imaging (MRI), positron emission tomography (PET), computed tomography (CT), and ultrasound (US), each carrying subsequent advantages and disadvantages. MRI appears to provide good spatial resolution, optimal tissue contrast, but with relatively low sensitivity, while PET compensates with its high sensitivity. MRI implies significant costs, contraindication in cases of pacemakers, claustrophobia, and risk to develop nephrogenic systemic sclerosis by the use of gadolinium [21]. To compensate for some of the limitations of these methods, combined strategies (such as PET/CT and PET/MRI) emerged. Nanoparticles can carry two or more imaging contrast agents, which provide complementary information obtained by multiplexed imaging [22]. They may be tracked by appropriate methods to obtain anatomical, physiological, and even molecular information [23]. Drug delivery can also be performed simultaneously [24]. 

Inorganic nanoparticles, such as gold, iron, or silica nanoparticles have unique physical properties, that derive from the properties of the material of origin. Iron nanoparticles, also known as superparamagnetic iron oxide nanoparticles (SPION) are composed of magnetite or maghemite (Fe_3_O_4_ and Fe_2_O_3_, respectively) and have superparamagnetic properties, thereby representing valuable contrast agents, in addition to their photothermal application and drug delivery potential [11]. SPION change the contrast properties of the tissues by modifying the local density of the protons and also by interfering with the local hydrogen protons, therefore altering the relaxation time T2, thus increasing the sensitivity of MRI imaging [25]. In comparison, gadolinium is a T1 contrast agent [5].

Gold nanoparticles are also valuable candidates for cardiovascular imaging due to their unique optical properties, reflected in surface plasmon resonance that lies at the base of significant light absorption and scattering. The size and shape of the nanoparticles modulate their function: particles smaller than 20 nm absorb the light, while the ones bigger than 80 nm trigger powerful light scattering [21]. Compared to iodine, gold offers 2.7 times more contrast, has higher absorption and prolonged circulation time and may ensure appropriate visualization of the circulatory system by CT scan with the advantage of venous injection over arterial catheterization [26]. Moreover, iodine is not appropriate for molecular imaging, since it cannot be functionalized [27]. Photoacoustic imaging allows the acquisition of real time details with improved tissue contrast compared to US, higher spatial resolution, and deeper depth than fluorescence imaging, without the use of ionizing radiation [28]. 

The recent advances in the field of nanomedicine applied on cardiovascular diseases concern the improvements of imaging detection, targeted treatments, preventive strategies, and the major steps to heart regeneration. These topics were systematically summarized in this review and included in two main sections: Heart remodeling and Heart regeneration. For each, we focused on the types of nanoparticles employed, functionalization strategies, and mechanisms by which they improved the treatment or diagnosis, aiming to shed light on the molecular pathways involved. 

## 2. Heart Remodeling

The human heart is composed mainly of cardiomyocytes (CMs), fibroblasts, and endothelial cells. CMs are the contractile units, providing the bulk mass of the myocardium, while endothelial cells are the major non-myocytic components [29]. Bilateral interactions between endothelial cells and CMs are very tight and are based on paracrine factors. The endothelium can modulate the function of CMs by producing nitric oxide, apelin, neuregulin-1, and thus modulating relaxation, hypertrophy, and cell proliferation. Similarly, CMs interact with endothelial cells primarily by producing vascular endothelial growth factor (VEGF), but also by fibroblast growth factor, hepatocyte growth factor, or angiopoietin-1. The communication mechanism through the direct cellular contact, mediated by the gap junctions, is added to the mentioned paracrine factors [30]. The administration of growth factors has multiple limitations: reduced half-life due to rapid degradation, limited diffusion through biological membranes and increased risk for toxicity. Nanoparticles may overcome these limitations by encapsulating the growth factor or the gene encoding the growth factor, ensuring a sustained release that allows the local activation of the specific pathway [31]. When the growth factors are used for the surface modifications of the nanoparticles, the targeting of its specific receptors may be achieved with secondary internalization of the nanostructure via endocytosis [32]. 

Aggression exerted on the heart, whether primary (genetic disorders, autoimmune myocarditis, and ischemic myocardial damage) or secondary (volume and pressure overload), triggers an adaptive response, which consists of concentric or eccentric ventricular hypertrophy, depending on the type of mechanical stress encountered (pressure or volume overload, respectively) [33]. The purpose of this modification is to ensure the optimal perfusion of the tissues to the new requirements of the body, given the increased preload and afterload [34]. When the heart’s ability to adapt is exceeded, the response becomes maladaptive, implying fetal gene expression, metabolic reprogramming, altered calcium metabolism, mitochondrial dysfunction, changing of sarcomere structure, insufficient angiogenesis, CMs death, and interstitial fibrosis [35].

The process through which the heart changes its volume, mass, and shape is called heart remodeling and the key steps contributing are hypertrophy, CMs loss, and interstitial fibrosis [36]. Following myocardial necrosis, the first key event is the establishment of inflammation, thus breaking the collagen fibers and implicitly the scaffold of the heart [37]. Tissue repair requires sustained angiogenesis that begins at the border zone and proceeds to the central core, with VEGF being one of the major proangiogenic molecules [38]. As the inflammation decreases, fibroblasts and myofibroblasts proliferate and deposit the extracellular matrix, a process controlled by the renin–angiotensin–aldosterone system [39]. Because nanoparticles are nanoscale structures, they have the potential to interfere at molecular levels and modulate these mechanisms. Therefore, multiple studies that target heart remodeling by nanoparticles have emerged, reporting improved imaging or delivery of drugs, proteins, or nucleic acid. Some of the strategies are summarized in the following subsections. 

### 2.1. Proteins of Interest

#### 2.1.1. Vascular Endothelial Growth Factor

The VEGF family encompasses six members: VEGF-A, VEGF-B, VEGF-C, VEGF-D, the orf virus VEGF (VEGF-E), and the placenta growth factor. Their effect is exerted by the activation of the specific receptors (VEGFR-1, VEGFR-2, and VEGFR-3) with tyrosine kinase activity. VEGFR-1 and VEGFR-2 are mainly expressed by endothelial cells, while VEGFR-3 is located on the lymphatic endothelium [40]. VEGF-A can bind to VEGFR-1 and VEGFR-2, but the effects of endothelial cell activation are mainly due to VEGFR-2 and consist in angiogenesis [41]. In comparison, VEGF-B was observed to sustain the viability of the vasculature rather than to stimulate novel vessel formation [42] and it also seems to preserve the myocardial mass and the contractility by VEGFR-1 activation, while enabling compensatory hypertrophy in myocardial infarction [43]. The mechanisms of crosstalk between endothelial cells and CMs are still missing, but it was revealed that the activation of VEGFR-2 in cardiac endothelial cells resulted in the paracrine release of epidermal growth factor receptor (EGFR) ligands, activating the growth cascade in CMs, and promoting myocardial hypertrophy with physiological features [44]. 

Considering the role of VEGF in angiogenesis, the prognostic of myocardial infarction may dramatically be changed by its local delivery, by enhancing neocapillary formation, and thus ameliorating the local ischemia and favoring the reduction in the necrotic area. Compared to bolus administration, the sustained release seems to be more beneficial, as VEGF toxicity due to increased plasma levels is prevented [38]. As clinical trials reveal, therapeutic effects are obtained at high doses, with the cost of significant side effects (hypotension, retinopathy, and malignant tumor progression [45]). The necessity to maintain an appropriate risk–benefit ratio, the short half-life and the rapid clearance are the reasons why VEGF requires a protective mechanism in the bloodstream, which can be provided by nanoparticles. The delivery of VEGF was achieved by multiple researchers (Table 1) through direct myocardial injection or intravenous release, by encapsulating or entrapping it in the nanoparticles.

Intravenous administration has the advantages of minimal invasiveness with concomitant sparing of the necrotic tissue by mechanical injury, while the main problem remains the low accumulation at the target site. Magnetic guided delivery may be a solution, as suggested by Zhang et al. [49]. However, in a clinical setting, there would be a significant risk of magnetic interference in patients with heart devices [51]; therefore, this strategy may be applied only in selected cases. In the vast majority of the studies, the local delivery of VEGF proved significantly increased capillary density, suggestive for angiogenesis. Because the newly formed vessels lack a basement membrane, the fluid leaks in the interstitium, with the formation of edema, promoting ventricular stiffness, which has a negative effect on heart function [52]. By stimulating lymphatic vessels formation through VEGF-C delivery, the edema liquid is drained and local conditions are improved [47].

Poly(lactic-co-glycolic acid) (PLGA) nanoparticles represent one of the most appealing polymeric nanoparticles for growth factor delivery because of their biocompatibility and biodegradability. They could ensure sustained release of VEGF throughout a month and significantly enhance tube formation and proliferation compared to free VEGF in vitro [38]. Human umbilical vein endothelial cells revealed a saturable uptake of the nanoparticles. The PLGA shell is degraded by hydrolysis into glycolic acid and lactic acid that are further metabolized by Krebs cycle [53], with local release of VEGF. However, the acidic microenvironment generated by PLGA degradation may induce protein denaturation [50]. Poly(ethylene oxide)-poly(propylene oxide)-poly(ethylene oxide) triblock copolymers are amphiphilic synthetic polymers that self-assemble into micelles in aqueous solution. The temperature-dependent gel formation by core-shell nanoparticles designed by Oh et al. [50] offer the additional advantage of regeneration support by offering an injectable scaffold, reflected in the greater left ventricle ejection fraction than the non-gel approach. The shape of the nanoparticle may also influence the duration of VEGF release, observing that star-shaped polyglutamic acid formulations discharged 30% of the total VEGF and linear ones only 17–18% on day 7 [46]. The main advantage of this formulation is the high encapsulation capacity (>99.9%) and the possibility to be embedded in hydrogel without angiogenic potential modification.

Targeting the VEGF-mediated pathway has immense potential on both imaging and treatment strategies. One main limitation is that the novel vessel formation is mainly assessed morphologically and functionally, while the activation of the specific pathways is not always assessed. In this regard, study should scrutiny the underlying molecular mechanisms to exclude confounding variables or to identify crosstalk. The nanoformulations presented here aimed to encapsulate VEGF, in order to enhance its local delivery. However, the potential of the nanomaterial to induce angiogenesis by itself should also be considered. Simultaneously imaging may also be performed in case of using magnetic nanobeads. Qiao et al. [47] used in vivo spectral imaging by labeling VEGF with a fluorescent dye. 

#### 2.1.2. Angiotensin II

Renin, produced mainly by the renal juxtaglomerular apparatus, cleaves the angiotensinogen, and generates angiotensin I, which is further converted by the angiotensin-converting enzyme into angiotensin II [54]. Angiotensin II plays a key role in the biology of the renin–angiotensin–aldosterone system, that may be targeted by nanoparticles to modulate heart remodeling (Table 2). 

Even though the activation of the renin–angiotensin–aldosterone system in the initial phases of cardiovascular diseases is compensatory, it becomes maladaptive. Angiotensin II controls myocardial remodeling (hypertrophy, fibrosis, and cell death), vascular remodeling, oxidative stress, and inflammation [62]. 

At least two classes of polypeptidic receptors are known to be involved in angiotensin II activity: AT1 and AT2. AT1 receptors are largely distributed in human tissues (blood vessels, brain, lungs, heart, kidney, and adrenal gland) and mediate the vast majority of the physiological effects of angiotensin II (aldosterone secretion, water and sodium absorption, vasoconstriction, and growth stimulation) [64]. The expression of AT1 receptors is augmented in experimental hypoxia and after myocardial infarction and its targeting by functionalizing the liposomes [63] or human serum albumin nanoparticles [56,57] with the specific aminoacidic sequence for the angiotensin II ligand allows the internalization by receptor-mediated endocytosis [56]. To test this approach in vivo, human serum albumin nanoparticles were designed to deliver milrinone (phosphodiesterase-3 inhibitor that increases the intracellular concentration of cyclin adenosine monophosphate and augment contractility and relaxation of the heart while decreasing the afterload [65]), a strategy that proved better pharmacokinetics (improved half-life, reduced clearance) and would permit a unique dose injection compared to nanoparticle-free formulation [57]. The main flaws of this design include relatively increased size, with subsequent risk of capillary obstruction and insufficient knowledge regarding the modulation of the side effects of milrinone. An anti-inflammatory response was simultaneously initiated, given the decrement of interleukin (IL) 6 and tumor necrosis factor (TNF) α in the myocardium, revealing the interconnected paths of heart remodeling.

AT2 receptors have antagonistic effects (vasodilation, inhibition of cell growth, and apoptosis) and are expressed during embryogenesis or in pathological conditions (ischemic heart disease, cardiomyopathies) [64,66]. The blockage of AT2 receptors enhances the hypertrophy of CMs and their overexpression prevents the increment of left ventricular mass index [54]. AT1 silencing, in the attempt to enhance AT2 function, may be achieved by gene silencing. Small interfering ribonucleic acids (siRNA) bring a substantial contribution in this field. They act at the post-transcriptional step by triggering the degradation of messenger ribonucleic acid and preventing protein expression [67]. Their intracellular delivery remains challenging, especially when the targets are found outside the monocyte–macrophage system. Dendrimers are branched polymeric nanoparticles with cationic ends that interact with anionic siRNA, forming a shield around the molecules and preventing their enzymatic digestion. The tadpole dendrimers tested by Liu et al. [62] were composed by a poly(amidoamine) head, which ensures the ability for endosomal escape and strong interactions with siRNA by high charge density and a tail represented by a cell penetrating peptide, that enhances the uptake by CMs. 

The beneficial effects of angiotensin-converting enzyme inhibitors, angiotensin receptor blockers, and renin inhibitors in cardiovascular diseases are well known and they seem to be augmented by targeted delivery by nanoparticles. In vivo ischemia-reperfusion injury served as an experimental design for irbesartan (AT1 receptor blocker) delivery by PLGA nanoparticles via intravenous injection, reaching threefold higher concentration in the ischemic tissue compared to the non-ischemic area [60]. Important accumulation was observed in the mononuclear–phagocytic system, with the inhibition of monocytes recruitment to the heart, providing an anti-inflammatory response that contributes to the reduction in the necrotic area 24 h after reperfusion, left ventricular dilation prevention, and improved left ventricular ejection fraction. 

The angiotensin-converting enzyme was observed to be elevated prior to the onset of myocardial infarction. Designing a targeting platform based on an angiotensin-converting enzyme inhibitor (lisinopril) and a nanoparticle with promising features as a contrast agent (gold nanoparticles) was reported by Ghann et al. [27], offering possible solutions for noninvasive monitoring of patients at high risk. Aliskiren (renin inhibitor) has poor bioavailability (2–7%), thereby high doses are required to achieve the desired effect. Polylactide acid nanoparticles reached aliskiren delivery, with additional blood pressure reduction compared to powder aliskiren and even increased nitric oxide synthase (NOS) heart activity, having the potential to reduce the dose and to lower the side effects [59]. 

An intermediary hydrolytic product of angiotensin I is angiotensin-(1-9), which was successfully delivered by a hybrid delivery system, based on polymeric nanoparticles and gold nanospheres, designed to inhibit cardiac hypertrophy [55]. As the addition of gold nanospheres did not have a cytotoxic effect, the proposed platform may be used for simultaneous treatment and imaging, benefiting from their optical properties [55]. Angiotensin-(1-9) is a counter-regulatory peptide of the renin–angiotensin–aldosterone system and acts through AT1 receptor activation [54]. Its exogenous administration is limited by the reduced bioavailability and half-life ensured by proteases degradation, therefore benefiting from a nanocarrier [55]. Liposomes are also effective for the delivery of angiotensin-(1-9) and its release may be enhanced by increased temperature (40–43 °C), preventing loss of bioavailability in the context of reduced release [58]. In this direction, the coating with the gold nanoclusters allowed near-infrared laser irradiation, with subsequent formation of nanopores and release of the content. 

#### 2.1.3. Apelin

The apelin receptor (APJ) is a G-protein coupled receptor with two natural ligands (apelin 13 and apelin 36) involved in cardiovascular homeostasis by promoting coronary and peripheral vasodilation. In experimental left ventricular hypertrophy or compensated heart failure, apelin, and APJ were maintained or downregulated, while their expression was reduced by the onset of decompensation [68]. Apelin administration was found to ameliorate not only ventricular hypertrophy but also glucose intolerance [69]. The poor in vitro and in vivo stability among the various isoforms of apelin was overcome by using PEG-coated liposomes as nanocarriers for [Pyr1]-apelin-13, a strategy that allowed the sustained release of apelin and ameliorated hypertrophy and fibrosis [70].

The synergistic effect of VEGF and apelin in promoting angiogenesis was emphasized by exposing human mesenchymal stem cells to PLGA nanoparticles, coated with VEGF DNA plasmid and loaded with apelin, which induced both endothelial differentiation and neovascularization [71]. Moreover, apelin sustained mesenchymal stem cells’ survival in a hypoxic medium, becoming a promising adjuvant for stem cell therapy, with implications in heart regeneration [72].

### 2.2. Inflammation

After myocardial infarction, inflammation is crucial for scar tissue formation. However, sustained or excessive inflammatory response turns into abnormal remodeling. The macrophages are recruited from the spleen by AT1 receptors and from the bone marrow by monocyte chemoattractant protein-1/CC Chemokine receptor 2 [73]. Moreover, chronic inflammation correlates with the progression of heart failure, pro-inflammatory macrophages being increased in the heart and the peripheral blood in these cases [74]. PLGA nanoparticles loaded with pitavastatin were injected intravenously as a preventive strategy in the myocardial mice model, and proved significant amelioration of the heart remodeling (ameliorated ventricle dilation and myocardial fibrosis) and reduced the recruitment of the splenic macrophages [75]. Considering the central role of the macrophages in cardiovascular diseases and the high accumulation of the nanoparticles in their cytoplasm, they may provide powerful tools for imaging applications (Table 3). Targeting the macrophages to reverse myocardial remodeling attracts the need to directly monitor them in vivo. Fluorodeoxyglucose (18F-FDG) is the most used radiopharmaceutic for PET imaging, but it lacks specificity for macrophages [76]. Nanoparticles may be engineered in the attempt to produce ideal radiopharmaceuticals for PET imaging, as proposed by Keliher et al. [77], who designed 5 nm polymeric nanoparticles labeled with 18F that exhibited high accumulation into macrophages and rapid renal elimination (6.5 min blood half-life in mice and 22.5 min in rabbits). 

SPION are divided in three categories, according to their size: oral (300 nm–3.5 µm), standard (50–150 nm), and ultrasmall SPION (<50 nm) [82]. To ensure the dispersion of SPION in aqueous media and prevent aggregation, a polymer coating is added. In case of coatings with molecular weights greater than 10 kDa, such as dextran or silicone, the affinity with the iron oxide core is poor and the adsorption reversible, resulting in clustering of multiple SPION and reduced predictability of clusters’ size [83]. Some other options are the adsorption of a dispersant with a molecular weight below 10 kDa, forming a core-shell structure [83] or the cross-linking of dextran [84]. Dextran-derived coating is usually preferred in case of SPION, with secondary internalization mainly in tissue-resident macrophages or monocytes, further lysosomal degradation to iron, and then following the physiological pathway of iron metabolism [85]. The uptake of SPION in macrophages is allowed by scavenger receptors (SR-A) [86]. In cells with high turnover, iron was not detected by Prussian blue staining or by T2 relaxometry after five to eight divisions [87]. Their limitations include complement activation and hypersensitivity reactions [84].

The advantages comprise an improved signal to noise ratio, the detection of subclinical changes in the atherosclerotic plaques, with potential effect in early detection and prevention of myocardial infarction and even immunomodulation surveillance in patients with heart transplantation, especially given the fact that it currently requires endomyocardial biopsy with specific risks [78]. However, SPION tracking by MRI is semiquantitative and the detection sensitivity is still 50 times lower compared to PET [76]. The magnetic properties may also favor the targeting and accumulation of a pharmacological agent at the desired site by coupling it with SPION and applying a magnetic field [85]. 

The inducible NOS (iNOS) has an increased expression in heart failure, in both CMs and infiltrating macrophages and it is accompanied by superoxide generation by macrophages, promoting oxidative damage. Macrophages expressing iNOS were reported to be key factors in myocardial remodeling, promoting oxidative stress, and fibrosis, thus representing potential therapeutic targets for immunomodulatory-based therapy [88] using nanoparticles (Table 4) with endogenous effect in this direction, as are cerium nanoparticles [89], or by the delivery of different molecules with anti-inflammatory or anti-oxidant effects.

The benefits of natural polyphenols include reduced reactive oxygen species production and endogenous antioxidant enzymatic activity in some cases, which may be potentiated by nanoparticles, considering their reduced bioavailability. Monomethoxypoly (ethylene glycol)-b-poly (dl-lactide) was used as a delivery agent for curcumin, in order to prolong its circulation time and enhance its biological function [95]. The inhibition of palmitate-induced CMs apoptosis was observed and one of the mechanisms is the activation of autophagy. The importance of this approach is high, given the prevalence of obesity and the association with cardiovascular diseases and high level of plasma free fatty acids.

## 3. Heart Regeneration

The human heart is known to have a limited regenerative capacity, with up to 50% of CMs being renewed throughout the life. It was reported that CMs’ turnover decreases from 1% by the age of 25 to 0.45% at age 75 [96]. Cytokinesis was observed only up to age 20 and was most prevalent in infants, providing strong evidence that CM proliferation is an integrative part of heart growth during childhood and adolescence [97].

The currently explored strategies to enable cardiac regeneration are cellular therapy, cardiac fibroblasts reprogramming and the stimulation of the residual CMs’ proliferation.

### 3.1. Cellular Therapy

Stem cells can differentiate into CMs by exposing them to factors involved in heart embryogenesis (activin A, bone morphogenetic protein-4, Wnt modulators) and can be delivered as dispersed or tissue-engineered cells [98]. The major flaws of stem-cell therapy are cell retention, engraftment, arrhythmias, and carcinogenesis, thereby limiting their clinical usage [99]. The main advantage of fetal CMs is the cardiomyocytic phenotype. They are also able to release VEGF and increase the local blood supply, but are limited by ethical debate, limited supply, and reduced survival [100]. The nanoparticle-based formulations are gaining attention, offering feasible solutions for stem cell delivery. The reduced survival of stem cells is partly due to inflammation and oxidative stress in the ischemic environment, which was ameliorated by the delivery of melatonin mediated by poly (lactide-coglycolide)-monomethoxy-poly-(polyethylene glycol) nanoparticles, with antioxidant effect [101]. Pretreatment of the adipose-derived mesenchymal stem cells with this nanostructure has led to improved effects with application in myocardial infarction. 

Nanofibrous scaffolds coated with collagen ensured an optimal environment for human mesenchymal stem cells differentiation to the cardiomyogenic line in vitro [102]. Desmin and GATA3 were expressed and intracellular calcium transport activity was detected. Sirtuin 6 (SIRT6) was increased and correlated with strong desmin-positivity, suggesting its powerful role in differentiation. The Wnt pathway was activated in SIRT6-depleted mesenchymal stem cells, impairing cardiomyogenic differentiation, which suggests its negative contribution to heart regeneration. 

Nanomedicine finds its place in the development of methods for tracking transplanted cells, so that both the success of the method and the side effects developed can be appreciated. In this sense, photoacoustic nanoparticles incorporating semiconducting polymers have allowed the detection of embryonic stem cell-derived CMs by photoacoustic imaging, without causing negative effects on cellular structures, while providing optimal spatial resolution [103]. Mesocellular foam silica nanoparticles are effective ultrasound contrast agents for human mesenchymal stem cells and the decoration of the nanosystem with superparamagnetic iron oxide nanoparticles results in concomitant magnetic resonance imaging performance [104]. By directly loading the iron oxide nanoparticles onto mesocellular foam silica nanoparticles, the pores were covered and, thus, the payload was decreased, concluding that the optimal method for the synthesis of the hybrid nanosystem was the in situ growing. The platform proved effective insulin-like growth factor delivery and proved amelioration of the left ventricle ejection fraction [104]. Küstermann et al. [105] also reported the usage of iron oxide nanoparticles for labeling of embryonic ventricular CMs by MRI. Marking the stem cells with 20 nm nanoparticles labeled with europium allowed them to be traced by ventriculography. They were administered in a collagen-rich vehicle, so that their retention in the area of myocardial infarction was facilitated, reducing their translocation to other organs [106].

In addition to the administration of stem cells as cell therapy, differentiated CMs can also be used, as reported by Nagase et al. [48], who used CM sheets concomitantly with poly(vinyl alcohol) fiber mats bearing PLGA nanoparticles for prolonged VEGF release for enhanced viability of the cells by angiogenesis or vascular maturation.

### 3.2. Cell Reprogramming

Cell reprogramming is another proposed approach for the induction of heart regeneration. Direct reprogramming represents the in situ conversion of the cells from one lineage to another, without an intermediate pluripotent state and with no need for cell expansion ex vivo [107]. So far, the limitations include: reduced conversion rate, possible depletion of starting cell population, lack of precise methods, identification of safe vehicles for reprogramming factors delivery [107]. Some of the advances in this field by the usage of nanoparticles will be further summarized.

Targeting the cardiac fibroblasts was achieved by functionalization of mesoporous silica nanoparticles with a ligand of tenascin-C, with the aim to deliver micro ribonucleic acid (miRNA) 1, 133, 208, and 499 [108]. Tenascin-C is a protein with high expression on the fibroblasts from the inflamed myocardium, being a powerful target for specific delivery. The used combination of miRNAs was previously reported to induce the direct conversion of cardiac fibroblasts to CMs [109]. Cardiac fibroblasts reprogramming into CMs was observed after one month of exposure, by morphologic changes (sarcomeric organization), protein synthesis (cardiac troponin T), gene expression (sarcomeric genes, specific transcription factor, and ionic channel), correlated with functional features (calcium transit) [108]. These results were also sustained by similar findings on an in vivo ischemia/reperfusion injury model.

Another strategy to achieve fibroblasts reprogramming is the delivery of transcription factors critical for heart development: GATA binding protein 4 (Gata4), myocyte enhancer factor 2C (Mef2CC), and T-Box transcription factor 5 (Tbx5) by gold nanoparticles conjugated with an arginine-rich peptide [110]. In vitro experiments performed on murine and human fibroblasts revealed acquired CM morphology, cardiac gene expression, and spontaneous contractility. In vivo myocardial infarction model provided evidence that the nanocarrier directly injected at the periphery of the necrotic area diminished the infarcted area, local fibrosis, and enhanced immunopositivity for Tbx5, α-Actin, cardiac troponin T, and α-myosin heavy chain. 

Transforming growth factor (TGF) β activation in fibroblasts leads to fibrosis and thus blocks cardiomyogenic differentiation [111]. The pH-sensitive delivery of a TGF-β inhibitor (SB431542) and a Wnt activator (CHIR99021) was successfully achieved by spermine-acetalated dextran-based nanoparticles, with improved biocompatibility through PEG coating and targeting the heart by atrial natriuretic peptide protein [111]. The delivery of the Wnt activator exhibited improved β-catenin staining in both the cytoplasm and the nucleus, while the delivery of TGF-β inhibitor proved increased cytoplasmic Smad3 stain.

### 3.3. Stimulation of Residual Cardiomyocytes

Several factors were found to stimulate the proliferation of the CMs, as IL-6, neuregulin-1, platelet-derived growth factor, fibroblast growth factor, or follistatin-like 1 [112]. The delivery of insulin-like growth factor and bromoindirubin-3-oxime was achieved by a hybrid hydrogel system based on their encapsulation in gelatin nanoparticles and further cross-linking with oxidized alginate, favoring not only the proliferation of CMs but also angiogenesis in areas of myocardial infarction [113]. Yes-associated protein 1 (YAP) and WW-domain-containing transcription regulator 1 (TAZ) are transcriptional coactivators [114]. The activation of Hippo/YAP pathway induces proliferation by translocation of YAP and TAZ from the cytoplasm to the nucleus, where they interact with transcription factors. By delivering TT-10 (a TAZ substitute) by PLGA nanoparticles, the proliferative effect on the CMs was prolonged and significant amelioration of the ejection fraction was observed [115]. The increased nuclear YAP stain sustained the activation of the Hippo/YAP pathway. 

EGFR family encompasses four transmembrane receptors with tyrosine kinase activity. EGFR2 lacks a specific ligand, being activated by heterodimerization with other members of EGFR family or by homodimerization [116]. The EGFR family controls many cellular processes—proliferation, differentiation, angiogenesis—being intensively studied especially for the favorable effect of its blockade in various neoplasms. Related to the exact effects in cardiovascular pathology, it has been associated with the regulation of blood pressure, endothelial dysfunction, atherogenesis, and cardiac remodeling, observing the association between an increased level of EGF ligands and the accelerated evolution of cardiovascular diseases [117]. Novel evidence also supports the role of EGFR2-mediated pathway in cardiac regeneration, performing as a key element in maintaining the function and the integrity of the heart [118]. 

Even though there are many nanosystems targeting the EGFR pathway with applications in neoplasia [119], they are lacking in the field of cardiology. Several researcher groups reported the use of microparticles instead. Acidic fibroblast growth factor and neuregulin-1 were delivered by PLGA microparticles in an experimental myocardial infarction rat model, with the ability to enable myocardial proliferation assessed by the increased number of Ki-67 expressing CMs in the necrotic and peri-necrotic area. The transient accumulation of cardiac progenitor cells was also reported [120]. The same agents were delivered by PLGA microparticles in a porcine model of ischemia-reperfusion lesion, with promising results [121]. Neuregulin-1 delivery by PLGA microparticles revealed an anti-inflammatory effect by the accumulation of CD206+ macrophages with a regenerative phenotype [122]. Neuregulin-1 is synthesized by endothelial cells and its receptors, EGFR2 and EGFR4 are activated by heterodimerization on the surrounding CMs. The regenerative effect is extendable by Wnt/β-catenin exposure [123]. Compared to nanoparticles, microparticles have several unwanted effects that make them less suitable for cardiovascular diseases: risk of embolization, aggregation, and reduced barrier crossing [124]. Strategies for targeting cardiac regeneration pathways are endless through nanomedicine, and the ability to modulate several such pathways simultaneously is promising in the treatment of heart disease.

## 4. Future Directions

**Figure 1:**
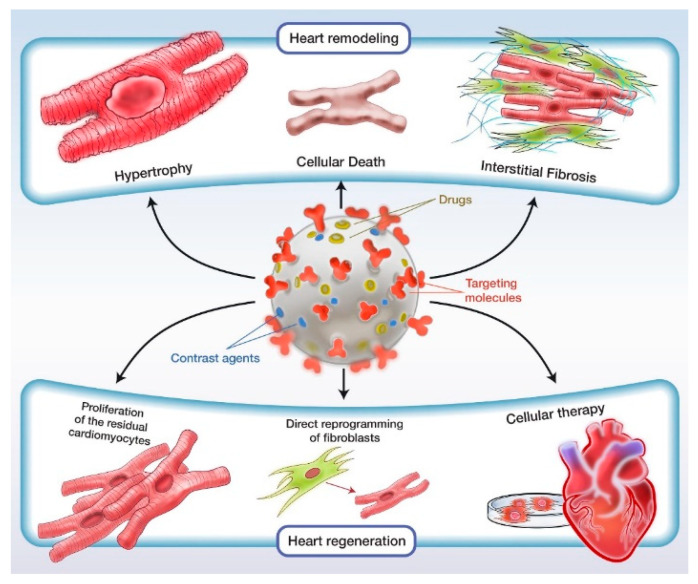
Application of nanoparticles in cardiovascular therapy and imaging.

New concepts emerge regarding the use of nanomaterials in the treatment, prevention, and diagnosis of cardiovascular diseases, especially in ischemic heart disease (Figure 1). By targeted delivery of the currently used pharmacological agents, but also by testing new ones, it seems that superior results can be obtained in vitro and in vivo. Knowledge of the molecular pathways involved in cardiac regeneration and remodeling shed light on new therapeutic targets. To translate these results into clinical practice, it is necessary to study the issues related to the adaption of the route of administration according to the end goal and context, interaction with the immune system, clearance and biodistribution, side effects on the cardiovascular system, but also on other systems. The future directions in this field derive from these limitations. The clearance of the nanoparticles is ensured mainly by the mononuclear phagocytic system, through which they are phagocytosed, digested, and excreted. One important limitation where the design of the study does not intend to target the macrophages is the inability of the nanoparticles to escape the mononuclear–phagocytic system. However, with appropriate functionalization, the nanomaterials may be delivered in any cell. There is a continuous need for fundamental studies that assess the impact of the size, shape, and surface decoration of nanotools because a rapid clearance associates with a reduced effect, while prolonged retention favors toxicity [125]. 

The identification of the side effects is another key parameter, which allows the formulation of contraindications and the continuous improvement of nano-mediated diagnostic and treatment strategies. Even though SPION seemed to be promising in the treatment and diagnosis of cardiovascular diseases, they promote myocardial injury by apoptosis, necrosis, pyroptosis, and ferroptosis through oxidative stress [126]. Several other nanoparticles have proved cardiac toxicity, such as zinc oxide [127] or nickel nanoparticles [128]. The avoidance of aggregation is also crucial, since it may induce thrombosis [27]. Extracardiac side effects are also numerous, requiring further studies to identify them according to the proposed nanodesign. One of the greatest concerns is the hypersensitivity reactions induced by complement activation, a case in which the rat experimental model only has limited value, thereby suggesting the need to develop appropriate experimental models [129]. 

With respect to the limited scope of our review, we summarized some of the main advances in the treatment of cardiovascular diseases by targeting the heart remodeling and regeneration by nanoparticles, emphasizing multiple successful encapsulation designs of currently used drugs or of newly emerging agents. We also emphasized how the versatility of nanomaterials allow them to be integrated in cardiovascular imaging, offering beneficial platforms to treat and monitor simultaneously.

## Figures and Tables

**Table 1:** VEGF delivery by nanoparticles.

Study	Year	Nanomaterial	Administration Route	Study Design	Outcome
O’Dwyer et al. [46]	2020	Hyaluronic acid hydrogel embedded with star-shaped polyglutamic acid polypeptides complexed with VEGF (400 nm measured by DLS and 200 by NTA)	N/A	In vitro drug release study	35 days of sustained VEGF release;
Qiao et al. [47]	2020	Crosslinked negatively charged heparin polysaccharide nanoparticle loaded with VEGF-A or VEGF-C (155 nm and 150 nm, respectively)	Intravenous injection	Acute myocardial infarction by left anterior descending artery ligation in mice	Delivery of VEGF-A enhanced angiogenesis, while delivery of VEGF-C triggered lymphangiogenesis and diminished local edema; Sequential administration resulted in improved heart function and reduced scar tissue;
Oduk et al. [38]	2018	PLGA nanoparticles loaded with VEGF (113 nm);	Injection into the peri-necrotic area	Immunocompromised NOD/SCID mice with left anterior descending coronary artery ligation	Improved vascular density, myocardial thickness, and decreased size of the necrotic area, independent on the dose; 31 days of sustained VEGF release;
Nagase et al. [48]	2017	PLGA nanoparticles loaded with VEGF (110.9 ± 12.0 nm)	N/A	Poly(vinyl alcohol) fiber mat incorporating nanoparticles loaded with VEGF for the transplantation of multilayered cardiomyocytes, implanted in the subcutaneous tissue of an athymic rat	After two weeks, the cardiomyocytes were still viable; The thickness of the sheet was preserved; Enhanced maturation of the blood vessels;
Zhang et al. [49]	2012	Magnetic nanobeads/adenoviral vectors-encoded *hVEGF* (300–600 nm)	Intravenous injection	Magnetic field guided treatment of experimentally induced acute myocardial infarction by left anterior descending artery ligation in rats	Increased hVEGF expression in heart at the luminal pole of the endothelial cells; Increased thickness of myocardium, the density of capillary vessels and decreased collagen deposition;
Oh et al. [50]	2010	270 nm nanoparticles with a core composed of lecithin and VEGF and a shell represented by poly(ethylene oxide)-poly(propylene oxide)-poly(ethylene oxide) triblock copolymer	Injection into the peri-necrotic area	The aqueous solution of nanoparticles was mixed with propylene glycol monocaprylate and, thus, a gel was formed at the site of myocardial infarction Acute myocardial infarction by left anterior descending artery ligation in rats	Significantly increased capillary density in the necrotic area with or without the gel formulation; The gel formulation did not change the arterial elastance (an indicator of heart compliance);

VEGF vascular endothelial growth factor; DLS Dynamic Light Scattering; NTA Nanoparticle tracking analysis; N/A not applicable; PLGA Poly(lactic-co-glycolic acid) nanoparticles.

**Table 2:** Targeting the renin–angiotensin–aldosterone system by nanoparticles.

Study	Year	Nanomaterial	Administration Route	Study Design	Outcome
Sepúlveda-Rivas et al. [55]	2021	Hybrid nanoparticles based on polymeric nanoparticles and gold nanospheres encapsulating angiotensin-(1–9) (sizes between 86.3 ± 1.3 and 108.1 ± 2.4)	N/A	In vitro norepinephrine-induced hypertrophy of CMs	No cytotoxic effect; The release of angiotensin-(1-9) peaks at 15 min; Prevented the hypertrophy of CMs;
Lomis et al. [56]	2021	215.2 ± 4.7 nm human serum albumin nanoparticles conjugated with angiotensin II peptide delivering milrinone	N/A	In vitro experiments on embryonic rat CMs (H9C2 line)	The nanomaterial has improved safety profile and reduced cytotoxicity compared to milrinone lactate; Significantly increased uptake by hypoxic and hypertrophic cells;
Lomis et al. [57]	2021	Human serum albumin nanoparticles conjugated with angiotensin II peptide delivering milrinone (sizes between 190.2 ± 5.7 and 245.6 ± 3.5 nm)	Intravenous or subcutaneous injection	Congestive heart failure induced by left anterior descending artery ligation in rat	Significantly improved the left ventricle ejection fraction at 24 h compared to nanoparticle-free formulation; After one hour the difference between these two approaches was not evident; The effect declined with no significant difference after 1 week of treatment; Intravenous administration was more effective than subcutaneous injections;
Bejarano et al. [58]	2020	190 nm thermosensitive liposomes encapsulating angiotensin-(1-9), coated with gold nanoclusters	Retrograde perfusion model	Ex vivo rat heart perfused with angiotensin-(1-9) after near infrared laser irradiation	No cytotoxic effect induced by the nanoplatform or by increased temperature; The biological effect of angiotensin-(1-9) was not affected; Improved heart function;
Pechanova et al. [59]	2019	279 nm polylactide acid nanoparticles loaded with aliskiren	Gavage	Spontaneously hypertensive rats	25% blood pressure reduction compared to 10% in the case of nanoparticle-free formulation; Increased NOS activity in the heart; Decreased the amount of collagen in the aorta;
Nakano et al. [60]	2016	200 nm PLGA nanoparticles incorporating irbesartan	Intravenous injection	Ischemia-reperfusion mouse model	The accumulation of the nanoparticles was observed only in the diseased tissue; The nanoparticle accumulated into CMs, monocytes and neutrophils; The concentration of irbesartan was 17-fold higher by nanovehicle delivery; Ameliorated the functions of the heart;
Hennig et al. [61]	2015	* Fluorescent core-shell quantum dots conjugated with angiotensin II	N/A	Rat mesangial cells, human adrenal gland carcinoma cells (NCI-H295R) and HeLa cells	Specific uptake of the nanoparticles by endocytosis in cell lines expressing AT1 (rat mesangial cells and NCI-H295R) was observed, with localization especially in the perinuclear region; Compared to pristine quantum dots, the proposed nanoparticles triggered a calcium influx into cytosol;
Liu et al. [62]	2013	Tadpole dendrimers conjugated with oligo-arginine for siRNA delivery to silence AT1 (sizes between 143 ± 29 nm and 247 ± 76 nm)	Intramyocardial injection	Ischemia-reperfusion rat model	At day 3, the expression level of AT1 mRNA was significantly reduced compared to control, with a slight tendency of AT2 level increment; The function of the heart was ameliorated and the necrotic area was reduced;
Ghann et al. [27]	2011	14.3 nm gold nanoparticles coated with lisinopril	#	Transmission electron microscopy evaluation of mice lung tissue	The nanosystem targeted the angiotensin-converting enzyme in the lung; The tendency to form large aggregates was observed;
Dvir et al. [63]	2011	142 ± 8 nm PEGylated liposome decorated with an aminoacidic sequence that targets the AT1 receptors (Asp-Arg-Val-Tyr-Ile-His-Pro-Phe)	Right jugular vein injection	Acute myocardial infarction induced by left anterior descending artery ligation in mice	About 50% of the CMs were targeted by the nanomaterial and around 80% under hypoxic conditions; The nanoparticles accumulate mainly in the left ventricle, while the healthy tissue exhibited only negligible accumulation;

N/A not applicable; CM cardiomyocyte; NOS nitric oxide synthase; PLGA poly(lactic-co-glycolic acid); PEG polyethylene glycol; AT1 angiotensin II receptor 1; siRNA small interfering ribonucleic acid; mRNA messenger ribonucleic acid; * Size not provided; # administration route not provided.

**Table 3:** Targeting macrophages for cardiovascular imaging.

Study	Year	Nanomaterial	Imaging	Purpose
Keliher et al. [77]	2017	5 nm polyglucose-based nanoparticles labeled with 18F;	PET	Imaging myocardial infarction and atherosclerosis;
Ueno et al. [78]	2013	20 nm iron oxide nanoparticles cross-linked with the PET isotope copper-64 and labeled with a fluorophore;	PET/CT	In vivo heart allograft imaging;
Majmudar et al. [76]	2013	13 nm dextran nanoparticles modified with desferoxamine and radiolabeled with zirconium-89;	Hybrid PET/MRI	Evaluation of inflammation in atherosclerosis;
Nahrendorf et al. [79]	2011	* Dextran-coated iron oxide nanoparticles labeled with fluorine-18 and a fluorophore;	PET/CT	Evaluation of inflammation in aortic aneurysm;
Morishige et al. [80]	2010	5 nm core composed by superparamagnetic iron oxide with a 10 nm dextran coat;	MRI	Evaluation of inflammation in atherosclerosis;
Lipinski et al. [81]	2009	125 nm lipid-based nanoparticles, loaded with gadolinium, targeting the macrophage scavenger receptor-B (CD36);	MRI	Atherosclerosis evaluation;

PET positron emission tomography; CT computer tomography; MRI magnetic resonance imaging; * size not provided.

**Table 4:** Reducing oxidative stress and inflammation by using nanoparticles in cardiovascular disease.

Study	Year	Nanomaterial	Study Design	Outcome
Jain et al. [89]	2021	Polycaprolactone blended with gelatin nanofibers decorated with 43 ± 5 nm cerium oxide nanoparticles	Phenylephrine-induced hypertrophy on neonatal primary cardiomyocytes	Reduced ROS; Prevent CMs hypertrophy;
Wang et al. [90]	2019	300 nm tanshinone IIA nanoparticles entrapped in hydrogel, ROS-sensitive	In vivo myocardial infarction induced by left anterior descending coronary artery ligation	Increased ejection fraction; Decreased necrotic area; Blocked inflammatory gene expression (IL-1β, IL-6, TNF-α)
Nabofa et al. [91]	2018	284.0 ± 17.9 nm poly (lactic acid) nanoparticles encapsulating curcumin and nisin	Isoproterenol induced myocardial infarction in guinea pigs	Prevented CMs necrosis by reduced ROS production; Reduced MPO activity;
Somasuntharam et al. [92]	2016	Gold nanoparticles functionalized with deoxyribozyme (14 ± 3 nm measured by TEM and 80 nm determined by dynamic light scattering)	In vivo myocardial infarction induced by left anterior descending coronary artery ligation	In vivo TNF-α silencing; Reduced iNOS, IL-12b, IL-1β and IL-6;
Niu et al. [93]	2011	* Cerium oxide nanoparticles	Oxidative stress induced in H9c2 cardiomyocytes by cigarette smoke extract	The nanoparticles prevented the oxidative damage by reduced ROS production with concomitant anti-inflammatory effect (inhibition of NF-κB activation and of inflammatory gene expression);
Niu et al. [94]	2007	7 nm cerium oxide nanoparticles	Transgenic mice with heart-specific expression of MCP-1	Reduced macrophage infiltration and pro-inflammatory cytokines; Reduced peroxynitrite formation; Ameliorated left ventricle dysfunction and heart remodeling;

ROS reactive oxygen species; CM cardiomyocyte; IL interleukin; TNF tumor necrosis factor; MPO myeloperoxidase; TEM transmission electron microscopy; iNOS inducible nitric oxide synthase; MCP-1 Monocyte chemoattractant protein-1; * size not provided.

## Data Availability

Not applicable.
